# Do cognitive and neurophysiological effects of acute memantine “challenge” predict its clinical benefits in Alzheimer’s Disease?

**DOI:** 10.1016/j.psychres.2025.116740

**Published:** 2025-09-22

**Authors:** Neal R. Swerdlow, Joyce Sprock, Christopher E. Gonzalez, Jenny Min Din, Jessica Minhas, Jo Talledo, Juan L. Molina, Yash B. Joshi, Gabriel C. Léger, Leslie Powell, Brinda Rana, Lisa Delano-Wood, Gregory A. Light

**Affiliations:** a Department of Psychiatry, School of Medicine, University of California, San Diego, La Jolla, CA, USA; b Department of Neurosciences, School of Medicine, University of California, San Diego, La Jolla, CA, USA; c VISN-22 Mental Illness, Research, Education and Clinical Center (MIRECC), VA San Diego Healthcare System, San Diego, CA, USA; d Moores Cancer Center, School of Medicine, University of California, San Diego, La Jolla, CA, USA; e VA San Diego Healthcare System, San Diego, CA, USA

**Keywords:** Alzheimer’s Disease, Auditory steady state response, Biomarker, Memantine, Mismatch negativity, Prepulse inhibition

## Abstract

“Personalized” interventions based on patients’ “biomarkers” may be valuable for treatments that benefit only subsets of patients. The NMDA antagonist, memantine, slows clinical progression of Alzheimer’s disease (AD); this effect is heterogeneous in magnitude and duration. This study tested whether acute cognitive or neurophysiological responses to memantine challenge predicted sensitivity to memantine’s therapeutic effects. Thirty individuals with mild-to-moderate severity AD (M:*F* = 13:17) and 24 comparably aged healthy subjects (HCS) (M:*F* = 12:12) were enrolled. Participants with AD were characterized on 9 experimental measures and their changes after acute memantine “challenge” (20 mg). We then assessed whether acute memantine effects on these measures predicted clinical change over a 24-week open-label trial of memantine. Baseline cognitive (Repeatable Battery for the Assessment of Neuropsychological Status) and neurophysiological measures (prepulse inhibition, P3a latency and auditory steady state response power and coherence) were impaired in participants with AD (p’s<0.05–0.0001); neurophysiological deficits were modestly reduced by acute memantine challenge. As a group, participants with AD showed no significant clinical changes across 24 weeks of memantine treatment; subgroups exhibited either small gains or deterioration. With one exception (mismatch negativity latency, *p* < 0.017), sensitivity of experimental measures to acute memantine challenge did not significantly predict clinical sensitivity to memantine. In summary, a challenge dose design identified neurophysiological measures sensitive to memantine in mild-to-moderate severity AD; acute memantine effects on one measure weakly predicted clinical outcomes over 24 weeks. Impairment in specific measures among participants with AD, and their opposition by memantine, might inform future efforts to identify treatment biomarkers in AD.

## 1. Introduction

Alzheimer’s disease (AD) is a neurodegenerative disorder affecting about 50 million people worldwide (Ali et al., 2015). Medications commonly used to treat AD symptoms include cholinesterase inhibitors (AChE-I’s) and the uncompetitive, low affinity N-methyl-d-aspartate (NMDA) antagonist, memantine (MEM). MEM is approved for treatment of moderate-to-severe AD, though it is often used in earlier stages (Areosa et al., 2005; Bakchine and Loft, 2008; Peskind et al., 2006; Pomara et al., 2007). Studies confirm good adherence to MEM as well as its ability to slow cognitive impairment in AD (Bakchine and Loft, 2008; Peskind et al., 2006; Bent-Ennakhil et al., 2017; Doody et al., 2007; Kavirajan 2009; Kishi et al., 2017a, 2017b); nonetheless, its clinical benefits are modest, short-lived and heterogeneous, with many patients showing little or no gains even after extended MEM trials.

It might be possible to enhance MEM’s efficacy by narrowing the target population, using laboratory-based biomarkers that identify MEM-sensitive subgroups (Light and Swerdlow 2015). A biomarker predicting MEM sensitivity might identify patients for whom MEM would be most beneficial; conversely, patients lacking a positive biomarker response might be counseled towards other treatments.

Prior work has shown that acute MEM challenge (20 mg p.o.) significantly enhanced laboratory measures of early auditory information processing (EAIP) (Swerdlow et al., 2016; Light et al., 2017; Molina et al., 2020) in patients with schizophrenia (SZ) and healthy comparison subjects (HCS). Measures of EAIP assess neurophysiological responses to structured sensory stimuli via electromyography (EMG) or electroencephalography (EEG) and are known to mediate neurocognition in SZ patients (Thomas et al., 2017). Using prepulse inhibition (PPI), mismatch negativity (MMN), gamma band auditory steady-state response (ASSR) and a metric of cortical “excitatory/inhibitory” (E/I) balance as dependent measures, a single 20 mg dose of MEM was associated with gains in these measures in both SZ patients and HCS, with effects ranging from small (MMN) to large (PPI) (Swerdlow et al., 2009, 2016; Light et al., 2017; Molina et al., 2020). The enhancing effects of MEM on these measures are consistent with findings by others in both HS (Korostenskaja et al., 2007) and in laboratory animals (Tikhonravov et al., 2010; Ma et al., 2015; Povysheva and Johnson, 2016; Swerdlow et al., 2024). Importantly, these responses to MEM challenge in humans demonstrated heterogeneity across subjects and were quantitative, so that the magnitude of “MEM sensitivity” could be calculated for each subject, based on differences between active and placebo doses. Conceivably, applying this “MEM challenge” approach to individuals with AD, in the span of two visits separated by one-week (see “Methods”), subgroups of AD patients might be stratified as “MEM-sensitive” vs. “MEM-insensitive”. This personalized medicine approach has been used in other fields (Hughes et al., 1990; Biller, 2007; Fruchter and Yigla, 2009), and attempted for some psychotherapeutics (Greden et al., 1981; Frodl 2017).

The feasibility of this 20 mg MEM “challenge dose” approach to identify biomarkers predictive of MEM sensitivity was tested in a small group (*n* = 6) of individuals with AD (Swerdlow et al., 2021). Subjects tolerated the 20 mg dose of MEM followed by cognitive and neurophysiological testing, as well as 24 weeks of open-label treatment with MEM (10 mg bid), without any serious adverse events. Based on the feasibility demonstrated in this small sample, we pursued findings in an expanded cohort of individuals with AD and gathered similar “baseline” cognitive and neurophysiological measures from a parallel group of comparable-age HCS. Our primary hypothesis was that the magnitude of the response of one or more experimental measures to acute MEM challenge would serve as a biomarker that predicts sensitivity of AD subjects to the clinical benefits of MEM over a 24-week trial.

## 2. Methods

### 2.1. Subjects

**Fig. 1.**
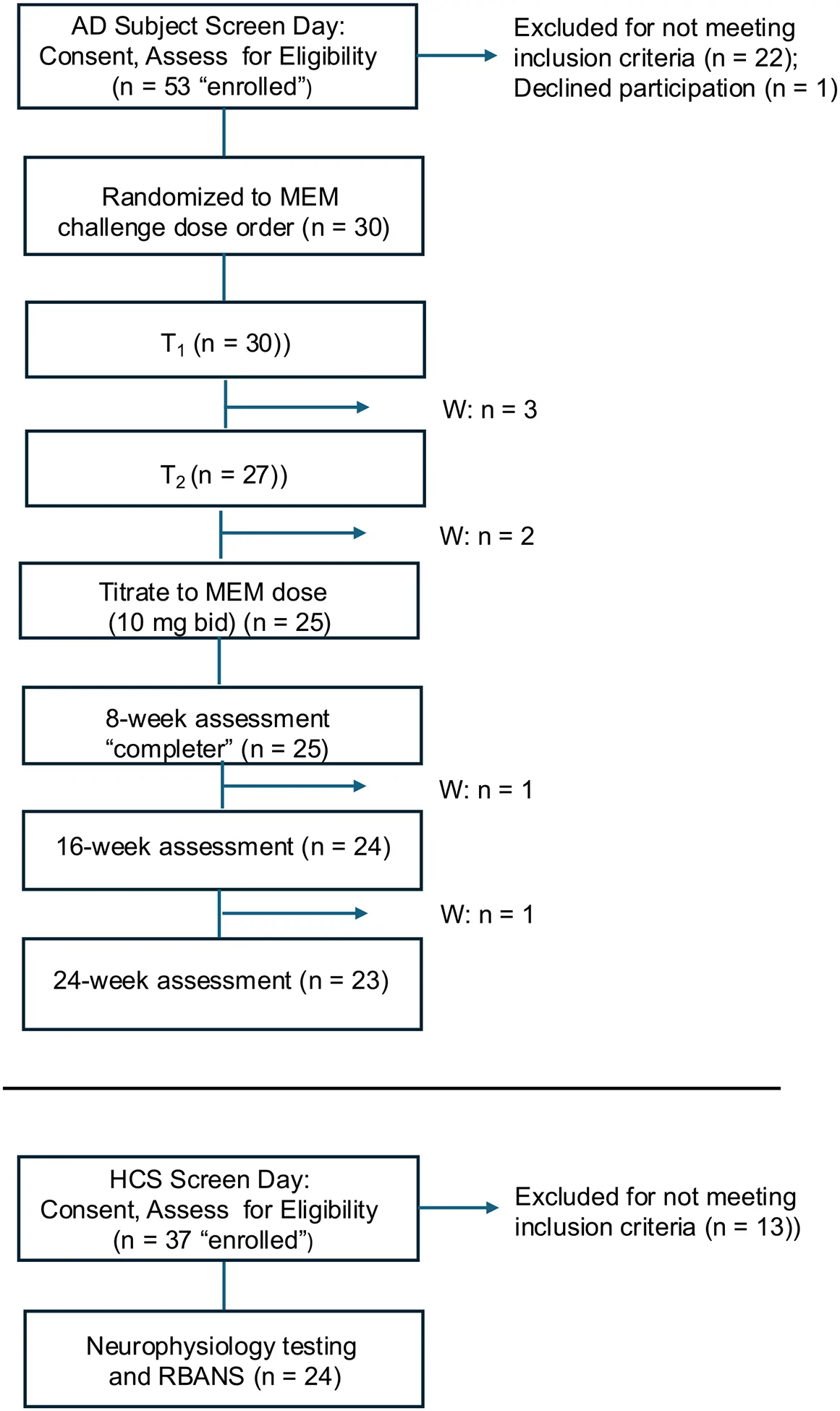
Consolidated Standards of Reporting Trials (CONSORT) diagram for subject “throughput” in the present study. Inclusion/exclusion criteria are seen in Table S1. Measure-specific exclusion criteria are found in Supplemental Methods.

This study (Fig. 1) was approved by the UCSD Human Subjects IRB (protocol #172,053). Participants ranged in age from 52–83 yo and met inclusion criteria as either AD subjects or HCS (Table S1). A total of 53 AD subjects and 37 HCS were initially screened for study participation, informed of the potential risks and benefits of the study and read and signed an IRB-approved consent form for study participation; of these, 30 AD subjects and 24 HCS were enrolled and completed at least one test day. The final “complete” study sample (see below) included 25 AD subjects (mean age (range) = 71.1 y (56–82); M:*F*4 = 11:14) and 24 HCS (mean age (range) = 65.0 y (52–81); M:*F*4 = 12:12). Of these 25 AD subjects, a subset (*n* = 6) had been characterized in our previous “feasibility” study (Swerdlow et al., 2021).

### 2.2. Procedures

For AD subjects, screening established a medical history, course and nature of cognitive and other symptoms, review of systems, current and past medications and included a physical examination, screening audiometry and electrocardiogram. Neuropsychological testing (Table S2) focused on measures of memory and executive function. The Montreal Cognitive Assessment (MoCA) and the Mini-Mental State Examination (MMSE) were acquired as inclusion criteria (Table S1: MoCA score 15–24 and/or MMSE score 10–22). The Alzheimer’s Disease Assessment Scale-Cognitive Subscale (ADAS-cog (70-point scale)) was acquired as the primary outcome measure; the Geriatric Depression Scale and Neuropsychiatric Inventory Questionnaire Severity Scale (GDS and NPI-Q) were acquired as secondary outcome measures. Blood was obtained for analyses of apolipoprotein-E genotype and specific genes (GRIN 2A, GRIN 2B, GRIN 3A) associated with MEM sensitivity in other neurologic conditions (Lesca et al., 2013; Li et al., 2016; Pierson et al., 2014). For HCS, screening evaluation included a structured clinical interview for psychiatric diagnoses (non-patient version), medical and substance history, urine toxicology, screening audiometry, the MoCA and MMSE.

For AD subjects, tests of acute MEM sensitivity followed approximately 7 and 14 days later (T1 and T2). Subjects arrived at 0800; after a brief review of symptoms, vital sign check and standardized breakfast, subjects ingested either placebo or MEM (20 mg) in a double blind, pseudorandomized balanced order. Vital signs and symptom ratings (100 mm visual analog scales (VAS) for “anxious”, “drowsy” and “happy”) were collected at regular intervals. The optimal dose and time-course for MEM effects on specific measures were selected based on past studies in HCS and schizophrenia patients (Swerdlow et al., 2009, 2016; Light et al., 2017; Molina et al., 2020).

At 210 min post-pill, acoustic startle and PPI were measured as previously described (e.g. Swerdlow et al., 2016). At 275 min post-pill, subjects completed the Repeatable Battery for the Assessment of Neuropsychological Status (RBANS) (Duff et al., 2008). At 345 min post-pill, EEG was acquired: specific measures included mismatch negativity (MMN) amplitude and latency, P3a amplitude and latency, auditory steady state response (ASSR) power and coherence, and excitatory/inhibitory balance (E/I Index) based on fronto-central (FC) recordings. HCS completed these same measures on one day, with no pill administration. These measures constituted the primary neurophysiologic (PPI, MMN amplitude and latency, P3a amplitude and latency, ASSR power and coherence and E/I Index) and neurocognitive (RBANS total score) tools for assessing both AD-related deficits compared to HCS, and acute MEM sensitivity. A list of primary and secondary outcome measures and experimental measures is seen in Table 1. Startle/PPI data from a subgroup of these AD subjects was reported previously (Swerdlow et al., 2023). Detailed methods for all neurophysiological measures are found in Supplemental Methods. After completing each test day, subjects “guessed” whether they received placebo or MEM, but were not given feedback.

Treatment Phase: Starting 7d after laboratory testing, AD subjects received MEM (5 mg at bedtime) with 5 mg added every 7d to a target dose of 10 mg (twice daily (bid)). MEM adherence was confirmed with weekly phone calls with caregivers and pill counts with prescription refills. One subject reduced her dose to 15 mg/d due to mild gait instability at 10 mg bid. Eight, 16 and 24 weeks after reaching target dose, subjects returned to the laboratory and were interviewed for health-related changes, primary (ADAS-cog) and secondary (GDS, NPI-Q severity) outcome measures were collected and a physical examination was conducted. The difference between baseline and 8-, 16- and 24-week values for outcome measures were the key metrics of MEM clinical sensitivity. Subjects were considered “complete” if they finished the acute MEM challenge phase of the study, titrated successfully to a steady dose of MEM for at least 8 weeks, and completed outcome measures after at least 8 weeks of MEM treatment. Previous studies have reported significant gains in ADAS-cog performance after 8 weeks of MEM treatment (Pomara et al. 2007).

### 2.3. Statistics

Four levels of statistical analyses were conducted:

First, “baseline” performance in the primary experimental measures was assessed using a between-subject rmANOVA to compare “placebo” day measures in AD subjects vs. measures in HCS; results were confirmed to be insensitive to pill order (i.e., placebo on T1 vs. T2) in the AD subjects. These analyses were used to identify measures where AD subjects were impaired compared to HCS and included all AD subjects who completed T1 and T2 (*n*4 = 27), and all HCS subjects who completed cognitive and neurophysiological testing (*n*4 = 24). Measure-specific criteria for exclusion are described in Supplemental Methods.
Second, the magnitude of “MEM effects” on subjective, behavioral, neurocognitive and neurophysiologic measures in AD subjects was assessed using rmANOVA with drug (placebo vs. MEM) as a within-factor. These analyses were used to establish which (if any) among the 9 primary experimental measures (RBANS total score, %PPI (60 ms), MMN amplitude and latency, P3a amplitude and latency, ASSR evoked power and inter-trial coherence, and E/I Index) exhibited a group-level MEM “signal”, i.e., change after acute MEM challenge, and included all AD subjects who completed T1 and T2 (*n*4 = 27).
Third, to test the primary hypothesis, measures of acute “MEM effect” were assessed as potential predictors of MEM clinical sensitivity. To accomplish this, MEM-induced changes in outcome measures (“change scores” from baseline vs. 8, 16 and 24 weeks of treatment for ADAS-cog, GDS and NPI-Q scores) were calculated in all AD subjects; positive numbers represented a clinical deterioration from baseline. Data were then submitted to: 1) rmANOVA using a median split based on the least vs. greatest acute MEM-effect (MEM minus placebo) in each experimental measure as a between-factor, and time (week) as a within-factor; and when possible, 2) simple regression of acute MEM effect vs. mean clinical “change score” for weeks 8, 16 and 24. In addition to median splits, alternative strategies for distinguishing “positive” vs. “negative” responders yielded similar results (e.g., subjects with ≥ 4-pt reduction vs. increase in ADAS-cog total score). For simple regressions, occasional outlier values (group mean ± > 3 SD) were excluded.
Fourth, exploratory analyses used ANOVA and simple regression to assess the potential predictive value of: 1) baseline (placebo) experimental measures; 2) MEM effects on additional experimental measures (e.g., autonomic and subjective responses to MEM challenge); and 3) potential genetic markers.

For the assessment of potential predictors of MEM sensitivity, to protect against family-wise error rates, comparisons were initially limited to MEM effects on the 9 primary experimental measures; for these analyses, alpha was set at 0.05. For exploratory analyses (with no strong *a priori* prediction), alpha was set at 0.01.

Two subjects completed acute MEM challenge (T1, T2) but exited the study after 8 (due to constipation) or 16 weeks (caregiver illness); a Last Observation Carried Forward design including these subjects did not substantively alter the results. Due to COVID-19 restrictions, some outcome testing was completed remotely. One subject was unable to follow instructions via teleconference during week 16 testing; those scores deviated significantly from scores obtained in-person at week 8 and week 24 and were thus replaced with the averaged scores for weeks 8 and 24.

## 3. Results

### Subjects:

AD (*n* = 25) and HCS (*n* = 24) demographics are seen ta. AD and HCS groups were roughly comparable in age, sex distribution and hearing threshold, but as expected differed significantly in MoCA (*F* = 91.04, df 1,46, *p* < 0.0001) and MMSE scores (*F* = 38.09, df 1,45, *p* < 0.0001). AD severity ranged from mild to severe based on MoCA scores (mean 16.4 (range 2 – 23)), with symptom duration averaging 3.1 years (range 0.6 – 6.6). AD subjects were college-educated, predominantly white (84 %), right-handed (92 %) non-smokers (100 %). Two-thirds of the AD subjects were taking an AChE inhibitor (*n* = 17); roughly three-quarters carried at least one APOE **ε4** allele.

### “Baseline” comparisons in AD vs. HCS groups (Fig. 2):

To identify measures in which AD subjects’ performance was abnormal, values from AD subjects’ post-placebo tests were compared to values from HCS. Post-placebo testing in AD subjects might have occurred on either T1 or T2, and analyses confirmed that there was no significant effect of test order on any measure. T1 vs. T2 correlations of the primary experimental measures are reported in Table S3.

**Fig. 2.**
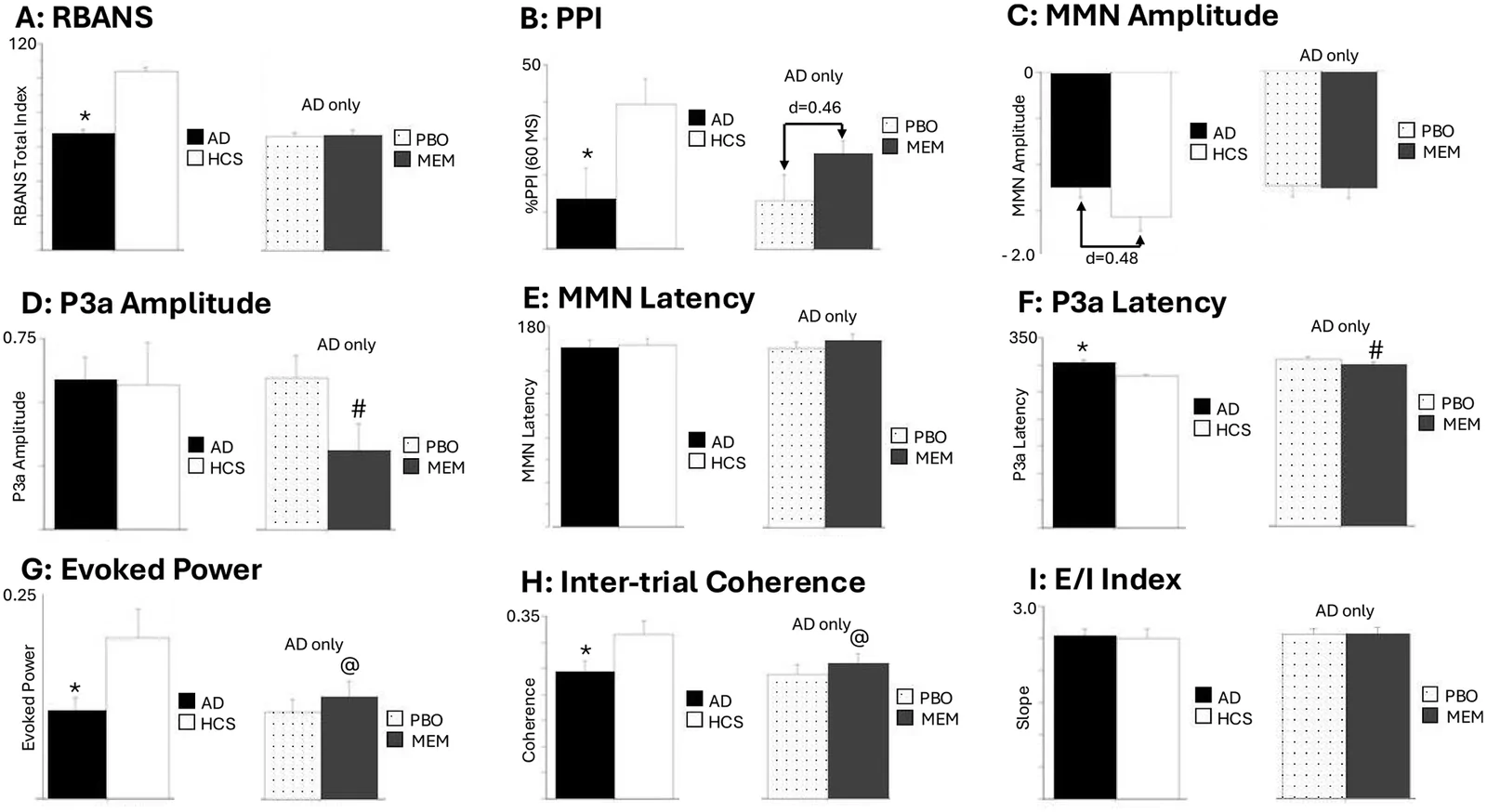
Nine primary experimental measures (described in Table 1) acquired at T1 and T2 from Alzheimer’s Disease (AD) subjects (post-placebo (PBO) and post-memantine (MEM)) and from Healthy Comparison Subjects (HCS; no pill). Shown for each measure (2A – 2I) are mean values (+ standard error of the mean (SEM)) from all subjects (left: AD subjects (PBO) vs. HCS (no pill)), and then values from AD subjects only (right: PBO vs. MEM). (*) indicates significant (*p* < 0.05) deficit in AD (post-PBO) vs. HCS (no pill). (#) indicates significant (*p* < 0.03–0.007) effect of pill (MEM vs. PBO) in AD subjects. (@) indicates trend (*p* < 0.08) towards significant effect of pill in AD subjects, for measures previously shown to be potentiated by MEM.

As expected, analysis of RBANS performance identified significant cognitive deficits in AD vs. HCS subjects, both for the Total Index Score (*F* = 84.18, df 1,51, *p* < 0.0001) (Fig. 2A) and across subscale domains (diagnosis: *F* = 74.15, df 1,51, *p* < 0.0001; domain: *F* = 74.15, df 4204, *p* < 0.0001; diagnosis x domain: *F* = 10.65, df 4204, *p* < 0.0001). The significant interaction of diagnosis x domain reflected the fact that deficits in AD subjects were most robust in immediate and delayed memory, and least robust in visuospatial, language and attention domains.

Measures detected no “baseline” difference between AD subjects and HCS in startle magnitude and habituation (diagnosis: *F* < 1; block: *F* = 68.09, df 1,51, *p* < 0.0001) or pulse alone startle latency (*F* = 2.28, df 1,42, ns) (not shown). Compared to HCS, AD subjects exhibited reduced PPI at the 60 ms interval (diagnosis: *F* < 1; interval: *F* = 6.07, df 4192, *p* < 0.0002; diagnosis x interval: *F* = 3.47, df 4192, *p* < 0.01; at 60 ms interval, diagnosis: *F* = 5.24, df 1,48, *p* < 0.027) (Fig. 2B).

EEG measures (Figs. 2C—H and Fig. S1) identified a trend towards reduced MMN amplitude (*F* = 2.93, df 1,49, *p* < 0.10; *d* = 0.48) but not latency (*F* < 1) in AD vs. HCS groups. In contrast, AD subjects exhibited abnormal P3a latency (slowed: *F* = 14.82, df 1,49, *p* < 0.0004) but not amplitude (*F* < 1) compared to HCS. AD subjects were also impaired in 40-Hz ASSR power (*F* = 5.68, df 1,45, *p* < 0.022) and coherence (*F* = 4.70, df 1,45, *p* < 0.036) compared to HCS. AD and HCS groups did not differ in baseline E/I Index (*F* < 1; Fig. 2I).

### “MEM effects” in AD subjects:

An acute dose of 20 mg MEM produced no significant changes in heart rate or subjective ratings of anxiety, drowsiness or happiness (Fig. S2). 60 % of the subjects correctly identified the pill that they ingested on the day that they received MEM, and 60 % correctly did so on the day that they received placebo.

Acute effects of MEM on the experimental measures are summarized in Fig. 2A–I. Test-retest (T1 vs. T2) correlations for most experimental measures was high (Table S3).

MEM had no significant acute impact on RBANS performance, either in the Total Index Score (*F* < 1) (Fig. 2A) or across domains (drug: *F* < 1; domain: *F* = 39.22, df 4, 204, *p* < 0.0001; drug x domain interaction: *F* < 1). Nonetheless, inspection of individual values of the “MEM effect” (MEM minus placebo) revealed substantial individual differences in acute MEM sensitivity of RBANS Total scores (Fig. S3).

We previously reported that MEM increased 60 ms PPI in a subgroup (*n* = 15) of the present AD cohort (Swerdlow et al., 2023). Analyses of the current, larger sample showed similar trends, but failed to achieve statistical significance. ANOVA of 60 ms PPI revealed no significant main effect of pill (*F* = 2.14, df 1,21, ns; *d* = 0.46; Fig. 2B). MEM had no significant effect on MMN amplitude or latency in AD subjects (F’s<1; Fig. 2C, 2E), but it did significantly reduce P3a amplitude (*F* = 8.84, df 1, 25, *p* < 0.0065; *d*4 = 0.56) and latency (*F* = 5.37, df 1,25, *p* < 0.03; *d*4 = 0.36) (Fig. 2D and F). MEM produced trends towards increased 40-Hz ASSR power (*F* = 3.52, df 1,23, *p* < 0.075; *d* = 0.23) and coherence (*F* = 3.33, df 1,23, *p* = 0.08; *d* = 0.21) in AD subjects (Fig. 2G and 2H), consistent with reports of small (d’s ≈ 0.2) but statistically significant changes in younger schizophrenia patients and HCS (Light et al., 2017; Swerdlow et al., 2024). Acute MEM had no significant effect on E/I Index in AD subjects (*F* < 1; Fig. 2I).

Post-PBO levels of PPI, P3a latency, ASSR power and ASSR coherence were all impaired in AD subjects compared to HCS (details above), but after MEM ingestion, none remained impaired relative to HCS among AD subjects (Fig. 2).

### Clinical changes over 24 weeks of open-label MEM treatment:

**Fig. 3.**
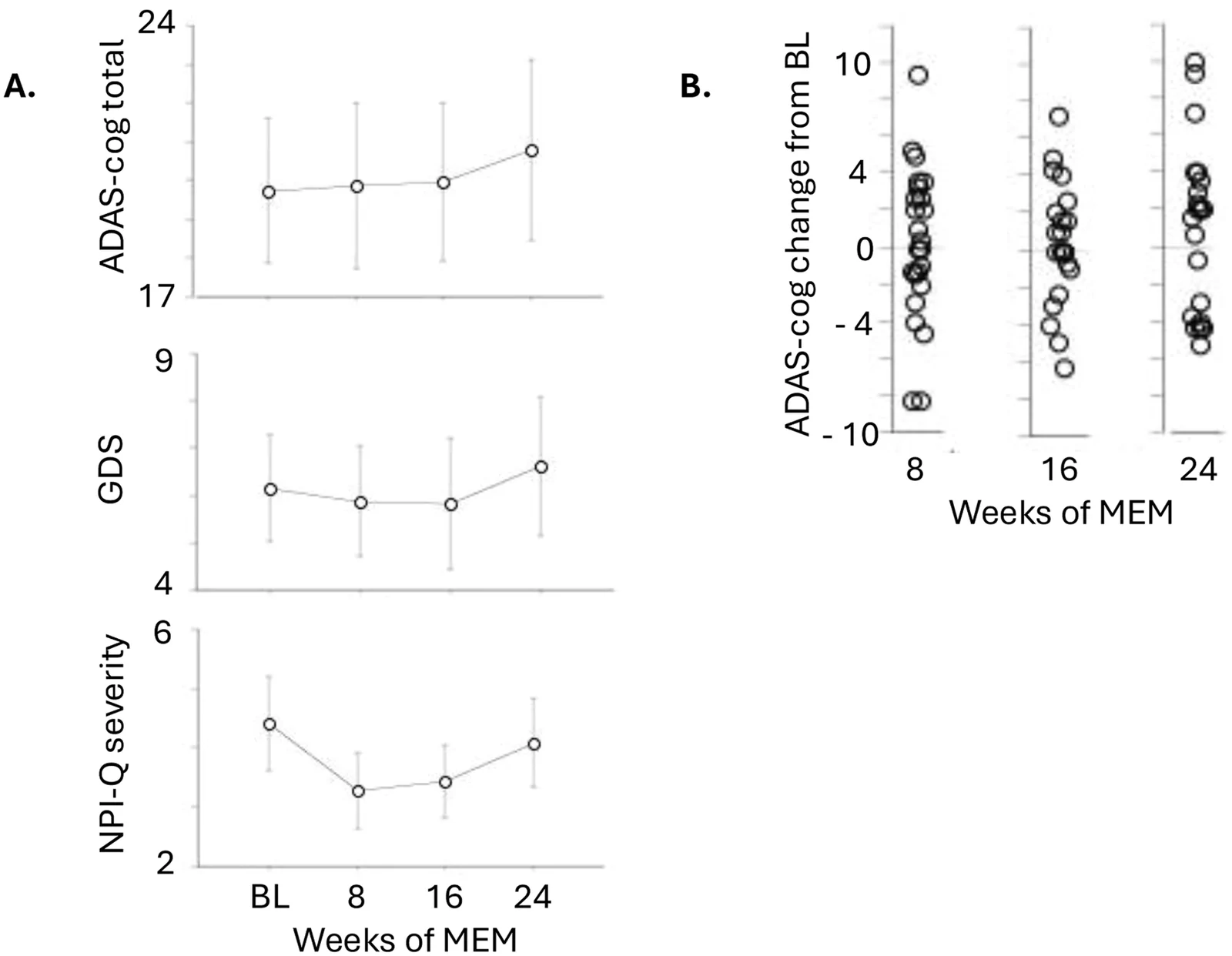
A. Primary (Alzheimer’s Disease Assessment Scale-Cognitive Subscale (ADAS-cog) total score) and secondary outcome measures (Geriatric Depression Scale (GDS) and Neuropsychiatric Inventory Questionnaire Severity Scale (NPI-Q) severity) in AD subjects at baseline (BL) and after 8, 16 and 24 weeks of MEM treatment (10 mg twice daily (bid)) (mean ± SEM). Group-level scores were relatively unchanged across the treatment phase. B. Distribution of change scores (treatment week minus BL) for ADAS-cog total score in individual AD subjects (each open circle), after 8, 16 and 24 weeks of MEM treatment.

AD subjects as a group exhibited little change in any of the 3 primary outcome measures (Fig. 3). ANOVA of ADAS-cog total scores revealed no significant effect of time (*F* < 1); means ranged from 19.77 (baseline) to 20.79 (week 24). Similar patterns were seen in GDS scores (*F* < 1; range from 6.23 (baseline) to 6.64 (week 24) and NPI-Q severity score (*F* = 1.37, df 3,63, ns; range from 4.27 (baseline) to 4.09 (week 24), with a nadir at week 8 (mean = 3.05). While the inclusive treatment group exhibited little clinical change, there was some heterogeneity in responses within this group. Using a 4-point change in ADAS-cog as “clinically meaningful”, 8 subjects exhibited meaningful cognitive decline over one or more time point of MEM treatment, and 8 subjects exhibited significant cognitive gains. At week 24, 5 subjects had ADAS-cog scores that were ≥ 4 points above baseline, and 5 subjects had ADAS-cog scores that were ≥ 4 points below baseline. *Put another way, after 6 months of treatment, roughly 80 % of the study sample had ADAS-cog scores that did not suggest clinically significant neurocognitive decline*.

### Primary “predictors” of MEM clinical sensitivity:

To test the primary hypothesis, analyses assessed the ability to predict sensitivity to MEM’s clinical effects based on acute MEM-induced changes in the 9 “primary” experimental measures: RBANS total score, %PPI (60 ms), MMN amplitude and latency, P3a peak amplitude and latency, ASSR evoked power and ASSR inter-trial coherence, and E/I Index.

**Fig. 4.**
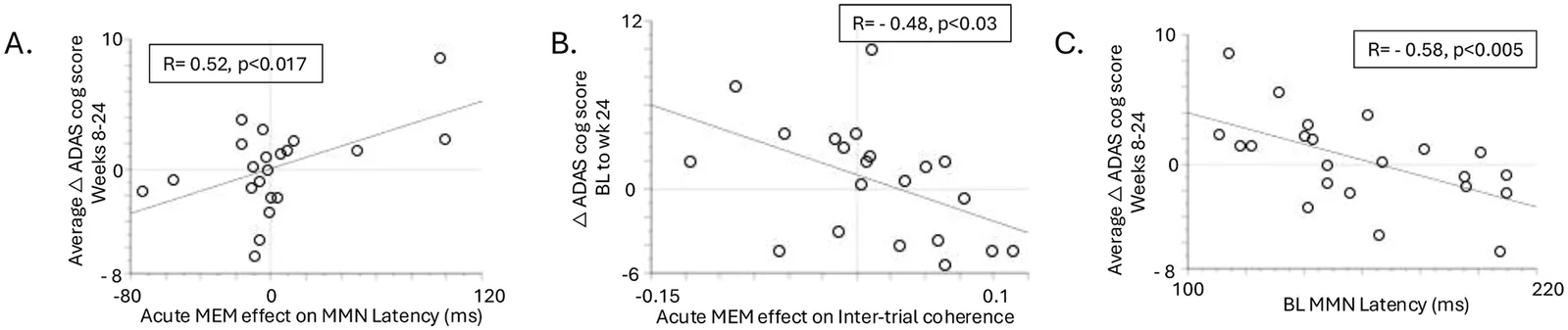
Significant “predictors” of sensitivity to the pro-cognitive therapeutics of MEM. A. Faster (reduced) Mismatch Negativity (MMN) latency after acute MEM “challenge” is associated with improved (reduced) ADAS-cog scores averaged across treatment weeks 8–24 (*r* = 0.52, *p* < 0.017). Similar effects were seen separately at weeks 8 (*r* = 0.53, *p* < 0.008) and 24 (*r* = 0.45, *p* < 0.04), missing significance at week 16 (*r* = 0.37, ns). B. Greater Auditory Steady State Response (ASSR) coherence after acute MEM “challenge” is associated with improved (reduced) ADAS-cog scores at treatment week 24 (*r*= −0.48, *p* < 0.03). C. Slower (more impaired) baseline MMN latency is associated with improved (reduced) ADAS-cog scores averaged across treatment weeks 8–24 (*r*= −0.58, *p* < 0.005); similar effects were seen separately at weeks 8 (*r*= −0.62, *p* < 0.001) and 16 (*r*= −0.56, *p* < 0.008), missing significance at week 24 (*r*= −0.35, ns). Regression terms for all measures are seen in Table 4 (for ADAS-cog) and Tables S5–6 (for GDS and NPI-Q, respectively).

As seen in Table 3 and Table S4, with rare exceptions, “MEM effects” on experimental measures did not strongly predict clinical gains in this inclusive AD cohort, as assessed by the primary and two secondary outcome measures. AD subjects were divided into two groups (“low” vs. “high” sensitivity) based on MEM effects on each of the 9 primary experimental measures, using median splits. In no case did changes in any of the 3 outcome measures across the 24 weeks of treatment differ significantly in these “low” vs. “high” sensitivity subgroups (Table 3). Simple regression analyses using ADAS-cog “change from baseline” at 8, 16 and 24 weeks of treatment (Table 4) largely confirmed this pattern, with two exceptions out of 9 measures. First, MEM effects on MMN latency correlated significantly with changes in ADAS-cog measures (reduced latency after acute MEM challenge was associated with cognitive gains with MEM treatment) after 8 weeks (*r* = 0.53, *p* < 0.008) and 24 weeks of treatment (*r* = 0.45, *p* < 0.04), with a similar trend at 16 weeks (*r* = 0.37, ns); this correlation remained robust when ADAS-cog gains were averaged across the 3 test days (*r* = 0.52, *p* < 0.017) (Fig. 4A). Second, MEM effects on ASSR coherence correlated significantly with changes in ADAS-cog measures after 24 weeks of treatment (*r*= − 0.48, *p* < 0.03; increased coherence after acute MEM was associated with cognitive gains with MEM treatment) (Fig. 4B).

With one exception out of 54 comparisons, MEM effects on experimental measures did not significantly predict (correlate with) changes in either GDS or NPI-Q severity scores after 8, 16 or 24 weeks of MEM (−0.33 ≤ *r*4 ≤ 0.45) (Tables S5–S6).

### *Exploratory analyses of potential predictors of MEM clinical sensitivity*:

Exploratory analyses assessed the potential predictive value of 1) baseline (post-placebo) levels of the 9 primary experimental measures; and 2) MEM effects on “secondary” experimental measures; and 3) potential genetic markers.

Across the 9 experimental measures, baseline performance in only a small number of measures correlated significantly ADAS-cog change at weeks 8, 16 or 24 (Table 4). Simple regression analyses using ADAS-cog change from baseline measures at 8, 16 and 24 weeks of treatment revealed significant correlations with baseline MMN latency at week 8 (*r*= − 0.62, *p* < 0.001) and week 16 (*r*= − 0.56, *p* < 0.008), with a similar trend at week 24 (*r*= − 0.35, ns). When ADAS-cog changes were averaged across weeks 8, 16 and 24, these values correlated significantly with baseline MMN latency (*r*= − 0.58, *p* < 0.005) (Fig. 4C); this effect is consistent with the outcome of the categorical “median split” rmANOVA of baseline MMN latency (*p* < 0.025; Table 3B). ADAS-cog changes at week 16 also correlated significantly with baseline P3a amplitude (*r* = 0.58, *p* < 0.005). Correlations of ADAS-cog changes with baseline P3a latency (*r*= − 0.53, *p* < 0.011) at week 16 missed significance based on an alpha set at 0.01 for these exploratory analyses.

Baseline (placebo) levels of experimental measures did not significantly predict (correlate with) changes in either GDS or NPI-Q severity scores after 8, 16 or 24 weeks of MEM (− 0.25 ≤ *r*4 ≤ 0.42) (Tables S5–S6).

MEM’s clinical impact did not differ based on age, sex, illness duration, genotype (APOe or glutamate receptor SNPs), concomitant use of an AChE inhibitor, baseline MoCA scores or other subject characteristics at study entry, nor was it significantly associated with MEM’s impact on measures of autonomic or subjective states (see Supplemental Results; Table S7).

## 4. Discussion

The goal of this study was to identify biomarkers that predict the clinical response to MEM over a 24-week trial in individuals with AD. The treatment regimen of MEM (10 mg bid for 24 weeks) is known to generate significant gains in, and delay deterioration of, the primary outcome measure – ADAS-cog total score – compared to placebo over a 24-week trial (Peskind et al., 2006; Pomara et al., 2007). While the present clinical trial was open-label, with no placebo comparison group, the fact that mean ADAS-cog scores remained relatively stable (did not rise significantly) over a 6-month period in AD patients suggests the likelihood that some patients experienced benefits from MEM, either via a slowing of their clinical deterioration or potentially even via “recovered” cognitive function. Indeed, at one or more time points in the present trial, 8 AD subjects experienced ≥ 4-point improvement in ADAS-cog scores compared to baseline levels (Fig. 3B) – a change thought to be “clinically meaningful” (e.g., Rockwood et al. 2010).

The primary crux of this study, however, was to determine whether it is possible to predict sensitivity to these benefits (or a lack thereof) based on the magnitude of response to an acute challenge with a single, high dose of MEM. Potential biomarkers were chosen, some of which had previously demonstrated group-level changes in response to acute MEM challenge. This “challenge” strategy has been used successfully to identify sensitivity to therapeutics for various psychiatric and neurologic disorders (Greden et al., 1981; Hughes et al., 1990; Biller, 2007; Fruchter and Yigla, 2009; Frodl 2017).

A group of healthy, age-comparable subjects was included in the study to identify which of the experimental measures at “baseline” were impaired in AD subjects. There is not a large literature on such measures in AD (see Supplemental Discussion), but in the present study -compared to HCS – AD subjects showed deficits in several measures of early auditory information processing, including PPI (at 60 ms), P3a latency, 40-Hz ASSR evoked power and inter-trial coherence, and, at a trend level (*d* = 0.48), MMN amplitude. This study was not designed to provide an in-depth parametric assessment of these complex experimental measures in AD; in fact, as it relates to the present study goals, it is not necessary for a measure to be deficient in AD in order for it to have predictive value for AD therapeutics. But if a neurobiological process is impaired in AD patients (as suggested by several measures seen in Fig. 2), this supports an inference that correcting that process might have clinical benefit.

Indeed, study findings provided suggestive evidence from some of these measures that MEM acted to partially “correct” these deficits after acute administration. MEM’s effects on PPI, P3a latency, ASSR evoked power and coherence were to “move” these measures closer to HCS levels; only some of these changes reached formal levels of statistical significance, but it is notable that: 1) several of these changes had previously been detected with comparable effect sizes in younger healthy and schizophrenia cohorts; MEM effects on ASSR power and coherence were previously reported to be age-dependent (inversely related to age: *r*’s= −0.71 and −0.63, respectively) and would thus be expected to be weaker in the present, older cohort (Swerdlow et al., 2016; Light et al., 2017); 2) in no cases did MEM exacerbate an existing deficit in AD subjects; and 3) after acute MEM ingestion, only one measure (P3a amplitude) remained significantly impaired vs. HCS. Of course, there are many reasons that the acute neurobiological response to a drug “challenge” – or a lack thereof - might not be relevant to the therapeutic potential of that drug administered chronically. In fact, if every patient exhibited a robust “corrective” response to a single dose “challenge”, this would limit the predictive potential for a drug that has a heterogeneous (variable) clinical profile. Still, the present findings of modest MEM effects in the “healthy direction” offered at least the possibility that the experimental heterogeneity in acute MEM effects might predict the therapeutic heterogeneity seen over 24 weeks of MEM treatment. However, direct tests with the experimental measures used here provided little support for such predictive power.

The most robust association between acute MEM “challenge” sensitivity and therapeutic response to MEM in this AD cohort came with a measure – MMN latency – that was neither impaired in AD subjects nor significantly impacted at a group level by acute MEM challenge (Fig. 2E). The robust correlation (*r* = 0.52) between acute MEM effects on MMN latency and the therapeutic response to MEM over 24 weeks of treatment suggests a shared heterogeneity in the response to MEM, i.e., that acute sensitivity of MMN latency to MEM challenge in AD subjects accounts for 33.5 % of the variance in the therapeutic response to MEM. But the lack of other obvious connections of MMN latency to either AD or MEM responsivity, in the context of 24 out of 27 “negative” predictive outcomes (Table 3A), raises the possibility that this association may be a chance event.

A dispassionate view of the primary findings of this study is that sensitivity to the effects of acute MEM “challenge” on 8 out of the 9 primary experimental measures does not robustly predict the therapeutic response to MEM in AD. There are several reasons that might account for these “negative” outcomes (see Supplemental Discussion) that largely reflect the limitations inherent to the present experimental design and sample. These findings do not in any way diminish the therapeutic value of MEM in AD, or the potential value of neurophysiological measures to serve as predictive biomarkers for such MEM effects; however, they do suggest that efforts to “personalize” this value through the use of a “challenge” design and predictive biomarkers should study measures other than those studied here, and/or apply approaches that generate combinatorial biomarker scores that may improve the sensitivity and predictive utility of neurophysiologic biomarkers. Meanwhile, the observed pattern of EAIP deficits in AD subjects may provide new clues for system-level dysfunction relevant to neurocognitive decline in AD.

## Supplementary Material

1

Supplementary material associated with this article can be found, in the online version, at doi:10.1016/j.psychres.2025.116740.

## Figures and Tables

**Table 1:** Outcome and experimental measures.

Outcome measures	
Primary:	Alzheimer’s Disease Assessment Scale–Cognitive Subscale (ADAS-Cog; 70-point maximum)
Secondary:	Geriatric Depression Scale (GDS) Neuropsychiatric Inventory–Questionnaire (NPI-Q) (Severity subscale)
**Experimental measures**	Repeatable Battery for the Assessment of Neuropsychological Status (Total Score) Prepulse inhibition of the acoustic startle response ( % PPI; 60 ms interval) Mismatch Negativity (MMN: amplitude (μV) and latency (ms)) P3a (amplitude (μV) and latency (ms))
Auditory Steady State Response	(ASSR: evoked power (μV^2^) and inter-trial coherence (gamma phase locking)) Excitatory/Inhibitory Index (slope)

**Table 2:** Subject demographics.

Diagnostic group	AD (*n* =25)	HCS (*n* =24)
Age (mean (range))	70.7 (56–81)	65.0 (52–81)
M:F	11:14	12:12
MoCA (mean (range))	16.4 (2–23)	27.1 (20–30)
MMSE (mean (range))	21.6 (10–29)	29.2 (24–30)
Education (mean y (range))	16.2 (12–22)	16.5 (12–20)
Hearing threshold (dB @ 1 kHz (mean (SD))	22.2 (11)	24.3 (8.6)
≥1 **APOE ε4 allele? (Y, N)**	*Y*4 = 18, *N*4 = 7	Not tested
Age at first symptoms (mean y (range))	68.1 (52.1 – 79.8)	NA
Years since first symptoms (mean (range))	3.0 (0.6 – 6.6)	NA
Acetylcholinesterase Inhibitor? (Y, N)	*Y* = 17, *N*4 = 8	NA
donepezil: galantamine: rivastigmine	11: 2: 4	NA

**Table 3:** Comparison of clinical gains (ADAS-cog) after 8, 16 and 24 weeks of MEM treatment in groups of AD subjects defined by: A. Low vs. high (median split) sensitivity to changes in experimental measures after acute “challenge” with MEM (20 mg); or B. Low vs. high (median split) baseline (BL) levels of these experimental measures.

A. Median split: low vs. high sensitivity of experimental measures to acute MEM challenge (“MEM minus baseline” difference score)									
Factor:	Median Split			Week			Split x Week		
Measure	F	df	p	F	df	p	F	df	p
RBANS total	1.36	1,20	ns	0.86	2,40	ns	0.05	2,40	ns
%PPI (60 ms)	1.63	1,16	ns	1.28	2,32	ns	1.52	2,32	ns
MMN amplitude	0.21	1,19	ns	0.51	2,38	ns	1.19	2,38	ns
P3a amplitude	0.31	1,19	ns	0.45	2,38	ns	0.17	2,38	ns
MMN latency	1.50	1,19	ns	0.43	2,38	ns	0.13	2,38	ns
P3a latency	2.27	1,19	ns	0.38	2,38	ns	0.90	2,38	ns
Evoked Power	0.13	1,18	ns	0.31	2,36	ns	0.28	2,36	ns
Inter-trial Coherence	0.19	1,18	ns	0.36	2,36	ns	3.14	2,36	ns
E/I Index	0.13	1,20	ns	0.89	2,40	ns	0.72	2,40	ns
B. Median split: low vs. high baseline levels of experimental measures									
Factor:	Median Split			Week			Split x Week		
Measure	F	df	p	F	df	p	F	df	p
RBANS total	0.79	1,20	ns	0.89	2,40	ns	0.75	2,40	ns
%PPI (60 ms)	0.26	1,16	ns	1.25	2,32	ns	1.09	2,32	ns
MMN amplitude	0.21	1,20	ns	0.51	2,40	ns	1.19	2,40	ns
P3a amplitude	0.31	1,20	ns	0.45	2,40	ns	0.17	2,40	ns
MMN latency	6.16	1,20	<0.025	0.89	2,40	ns	0.66	2,40	ns
P3a latency	2.27	1,20	ns	0.38	2,40	ns	0.90	2,40	ns
Evoked Power	0.62	1,18	ns	0.38	2,36	ns	4.01	2,36	<0.03
Inter-trial Coherence	0.12	1,18	ns	0.62	2,36	ns	3.35	2,36	<0.05
E/I Index	0.25	1,20	ns	1.23	2,34	ns	8.68	2,40	<0.0008

**Table 4:** Correlations (r) of acute MEM (20 mg) effect on experimental measures (A) or baseline measures (B). vs. change in ADAS-cog score after weeks 8, 16 and 24 of MEM treatment (10 mg bid).

A. MEM effect on:	Wk 8 minus BL	Wk 16 minus BL	Wk 24 minus BL
RBANS total score	0.20	0.32	0.21
%PPI, 60 ms interval	−0.13	−0.01	−0.17
MMN amplitude	−0.25	−0.21	−0.26
P3a amplitude	−0.12	−0.34	−0.12
MMN latency	**0.53 (*p* < 0.008)**	0.37	**0.45 (*p* < 0.04)**
P3a latency	0.24	0.34	−0.14
Evoked power	0.23	0.11	−0.34
Inter-trial coherence	−0.05	0.03	**−0.48 (*p* < 0.03)**
E/I Index	0.14	0.02	0.01
**B. Baseline levels of:**			
RBANS total score	−0.25	−0.18	−0.34
%PPI, 60 ms interval	0.00	−0.12	−0.13
MMN amplitude	0.38	0.00	0.19
P3a amplitude	0.23	**0.58, *p* < 0.005**	0.02
MMN latency	−**0.62, *p* < 0.001**	−**0.56, *p* < 0.008**	−0.35
P3a latency	−0.40, *p* = 0.05	−0.53, *p* < 0.011	−0.27
Evoked power	−0.26	0.21	0.35
Inter-trial coherence	−0.19	0.16	0.29
E/I Index	−0.01	0.33	−0.34

**BOLD** = meets pre-set levels of statistical significance.
