# Supplementary material for: Do cognitive and neurophysiological effects of acute memantine “challenge” predict its clinical benefits in Alzheimer’s Disease?
Source: Psychiatry Res. Author manuscript; Available in PMC 2026 Jun 13. (PMC13263901; doi:10.1016/j.psychres.2025.116740)
Supplement: 1 [file NIHMS2183330-supplement-1.docx]

Supplemental Methods, Results and Discussion for:

**Do cognitive and neurophysiological effects of acute memantine “challenge”**

**predict its clinical benefits in Alzheimer’s Disease?**

Neal R. Swerdlow, MD, PhD^1,3^, Joyce Sprock, BA^1,3^, Christopher E. Gonzalez, PhD^3^, Jenny Min Din, BS^1^, Jessica Minhas, BS^1^, Jo Talledo, BA^1^, Juan L. Molina, MD^1,3^, Yash B. Joshi, MD, PhD^1,3^, Gabriel C. Léger, MD^2^, Leslie Powell, MS, RN, AGPCNP ^2^, Brinda Rana, PhD^4^, Lisa Delano-Wood, PhD^1^, Gregory A. Light, PhD^1,3^

1. Dept. of Psychiatry, School of Medicine, University of California, San Diego, La Jolla, CA
2. Dept. of Neurosciences, School of Medicine, University of California, San Diego, La Jolla, CA
3. VISN-22 Mental Illness, Research, Education and Clinical Center (MIRECC), VA San Diego Healthcare System, San Diego, CA, USA
4. Moores Cancer Center, School of Medicine, University of California, San Diego, La Jolla, CA

**1. Supplemental Methods:**

1.1. Subject inclusion / exclusion criteria for Alzheimer’s Disease (AD) and healthy comparison subjects (HCS) groups are seen in Table S1:

**Table S1. Inclusion and exclusion criteria**

Inclusion criteria (All subjects):

1. Age 50-83

2. Medically stable

3. Hearing threshold < 45 db(A) at 1000 Hz

4. Ambulatory

5. Can provide informed consent

6. Can tolerate laboratory testing

Added inclusion criteria for AD subjects:

1. Documented clinical diagnosis of AD

2. MoCA score 15-24 and/or MMSE score 10-22

3. Accompanied by knowledgeable historian

Added inclusion criteria for HCS

1. MoCA score >24 and MMSE score >22

Abbreviations: TBI = traumatic brain injury;

TIA = transient ischemic attack;

AChE = acetylcholinesterase

Exclusion criteria (All subjects):

1. History of TBI, brain tumor or seizure disorder

2. Active systemic illness (e.g., heart disease, liver failure, renal insufficiency, cancer, HIV, tuberculosis, Hepatitis C)

3. Current psychiatric or neurologic illness (other than AD for AD group)

4. History of vascular disease, myocardial infarction, cerebrovascular accident, TIA

5. Substance dependence

6. Current medications include pro-cognitive agent

7. Positive urine toxicology for non-prescribed psychoactive substance

Added exclusion criteria for AD subjects:

1. Current or past treatment with MEM or amantadine

2. Previously unable to tolerate AChE-inhibitors

1.2. Neuropsychological tests conducted on screen days, and group mean (SD) scores, are seen in Table S2. For most measures, AD subjects averaged 1.0 – 2.5 SD’s below age-matched normed values.

**Table S2.** Neuropsychological Screening Assessment

| **Neuropsychological Test** | **Mean (SD)** | **Impairment** |
| --- | --- | --- |
| Boston Naming Test (T-Score) | 38.09 (13.01) | Mild |
| CVLT* Trials 1-5 (T-Score) | 26.39 (11.34) | Moderate |
| CVLT* Long Delay Free Recall (T-Score) | 26.37 (8.70) | Moderate |
| CVLT* Recognition Discriminability (T-Score) | 24.07 (9.55) | Moderate-to-Severe |
| D-KEFS* Trail Making Test Switching (T-Score) | 35.21 (13.16) | Mild |
| D-KEFS* Verbal Fluency - Letter (T-Score) | 45.45 (14.64) | ---- |
| D-KEFS* Verbal Fluency - Category (T-Score) | 34.78 (11.88) | Mild-to-Moderate |
| D-KEFS* Verbal Fluency - Switching (T-Score) | 30.76 (13.21) | Mild-to-Moderate |
| WMS-IV* Logical Memory 1 (T-Score) | 30.56 (10.29) | Mild-to-Moderate |
| WMS-IV* Logical Memory 2 (T-Score) | 23.04 (6.67) | Moderate-to-Severe |
| WMS-IV* Logical Memory Recognition (Raw Score) | 14/30 (-2.1) | Moderate-to-Severe |

*CVLT = California Verbal Learning Test

*D-KEFS = Delis-Kaplan Executive Functioning System

*WMS-IV = Wechsler Memory Scale 4th Edition

1.3. Methods for the primary experimental measures and measure-specific exclusion criteria follow below. Additional details can be found in published reports (Light et al. 2017; Molina et al. 2020; Swerdlow et al. 2016).

Experimental measures were timed to coincide with peak blood levels of MEM after a single oral dose of 20 mg (Sonkusare et al, 2005). Startle testing began 210 min after pill administration, RBANS testing followed at 385 min post-pill, MMN testing commenced 345 min post-pill and ASSR measures followed immediately after MMN testing, beginning about 385 min post-pill.

1.3.1. Acoustic startle response and prepulse inhibition (PPI): Startle was measured as previously described (e.g., Swerdlow et al. 2016). Broadband noise (70 dB(A)) preceded active stimuli by 3 min and persisted as a background noise during the test. The session consisted of 42 trials, with six conditions: a 115-dB(A) 40-ms noise burst (pulse alone) and the same burst preceded 10, 20, 30, 60, and 120-ms by a 20-ms prepulse 15 dB above background; using 15-dB prepulses with this startle system, prepulse-associated EMG activity is <0.5% of startle stimulus-induced levels. To measure startle habituation, three pulse-alone trials were presented at the session beginning (Block 1) and end (Block 3). During active and placebo drug tests, startle ‘non-responsiveness' was defined by a mean startle magnitude < 5 units on pulse alone trials; this resulted in the exclusion of 7 subjects (AD = 5 (1 after PBO, 4 after MEM); HCS = 2). %PPI was defined as:

(100−(100 × magnitude on prepulse trial/magnitude on pulse alone trials)).

%PPI for the 60-ms prepulse interval was selected as the primary PPI measure, based on prior findings that this interval is most sensitive to MEM effects in AD subjects (Swerdlow et al. 2023), and the present findings that it is most sensitive to PPI deficits in AD subjects vs. HCS (Figure 2B).

1.3.2. Mismatch negativity (MMN): Auditory stimuli were presented at 85 dB SPL via Etymotic ER3-A insert earphones. A three-tone auditory oddball paradigm comprised of 85% standards (50-ms, 1000-Hz) and 15% deviant stimuli (7.5% per deviant type) that differed from the standard in duration (125-ms tones at 1000-Hz), or both pitch and duration (125-ms tones at 1100-Hz). A pseudorandomized sequence ensured that a minimum of three standard tones were presented between each deviant stimulus. All tones had 5-ms rise/fall times and were presented with a fixed 500-ms stimulus onset asynchrony. Total recording time was ~45 min per session. Subjects were instructed to ignore auditory stimuli while viewing a silent movie.

EEG data were continuously recorded at a sampling rate of 2048-Hz from 64 channels using a BioSemi ActiveTwo system (www.biosemi.com) (for details of electrode configuration and offline processing in EEGLAB, see (Greg’s citation here)). Briefly, data were downsampled to 1024-Hz and digitally filtered using a 0.5-Hz high pass and 60-Hz notch filter to remove slow drifts and line noise, respectively. Bad channels were detected using PREP plugin (Bigdely-Shamlo et al., 2015) and interpolated. Large amplitude deviations (e.g., data points exceeding +300 mV and 2500ms point spread threshold) were removed using the ‘trimOutlier’ plugin prior to Infomax ICA decomposition. Independent components maximally contributing to eye blink artifacts were detected with the ‘IC Label’ plugin (Pion-Tonachini et al., 2019) (IC probability > 0.7) and were subsequently removed from the data.

Continuous data were segmented at -150 – 450 ms relative to the onset of auditory stimuli. Deviant-minus-standard difference waves were generated for each deviant type and low-pass filtered (30-Hz; Hamming window, transition bandwidth=1). MMN and P300 were computed as the mean amplitude across the 110–210 ms and 250-350 ms time windows, respectively; from the grand average difference waveform at electrode Fz.

1.3.3. Auditory Steady State Response (ASSR): The ASSR paradigm utilized 1 ms, 85 dB clicks presented in 500 ms trains at a frequency of 40 Hz. A total number of 250 click trains were played with an inter-train interval of 0.5 s. The auditory stimuli were delivered through insert earphones. Participants were instructed to ignore auditory stimuli while viewing a silent movie. The ASSR recording lasted approximately for 4 min. Subjects unable to complete at least 75 click trains (AD = 1; HCS = 1) were excluded from analysis. Continuous data from the Fz electrode were segmented -1000 to 1000 ms relative to the stimulus onset.

Gamma evoked power and phase locking were calculated on wavelet coefficients obtained from Morlet wavelet transformation of the segmented data (representing the 5 to 100 Hz frequency range, with a total of 96 frequency layers). Gamma inter-trial phase locking quantifies the consistency of the oscillatory phase across individual trials, ranging from 0 (purely non-phase-locked activity) to 1 (fully phase-locked activity). For statistical analyses, mean values from the 200 to 500 ms post-stimulus time window were extracted from the 40 Hz frequency layer.

1.3.4. Excitatory/Inhibitory (E/I) Index: Auditory stimuli from the oddball paradigm were used to calculate the E/I index. EEG signals (i.e., 500 ms post-stimuli epochs) were extracted from 9 fronto-central electrodes (Fz, Cz, CPz, F1, F2, C1, C2, CP1, CP2) and were averaged across stimuli. Time-domain signals were then decomposed into their frequency-domain components via power spectral density (PSD) estimation using Welch’s method. PSDs from the 1–45 Hz range were used to characterize the aperiodic “background” or 1/f-like signal using the ‘fooof’ algorithm (Donoghue et al., 2020).

**2. Supplemental Results:**

**2.2.** EEG responses in HCS and AD subjects after placebo (PBO) or MEM are seen in Figure S1.

**Figure S1.** A. MMN and P3a in HCS, AD subjects during placebo and 20 mg MEM, and Grand Averages. X-axis indicates time in ms and Y-axis indicates amplitude (microvolts). A trend of reduced MMN amplitude (p<0.10) but not latency was observed in AD vs. HCS groups. AD subjects exhibited abnormal P3a latency (slowed: p<0.0004) but not amplitude compared to HCS. MEM had no significant effect on MMN amplitude or latency in AD subjects, but it did significantly reduce P3a amplitude (p<0.0065) and latency (p<0.03). B. Time-frequency plots in HCS, AD subjects during placebo and 20 mg MEM, and MEM-placebo difference. X-axis indicates time in ms and Y-axis indicates frequency. Top row: Evoked Power (EP). Color indicates power (microvolts squared). Bottom row: Inter-trial coherence (ITC). Color indicates phase locking factor (“coherence”). Compared to HCS, AD subjects exhibited EP and ITC deficits after PBO, but not after MEM; in AD subjects, compared to PBO, MEM was associated with trends towards increased power (p<0.075) and coherence (p=0.08).

**Figure S2.** Changes in heart rate and subjective ratings of anxiety, drowsiness and happiness (mean + SEM), in AD subjects after ingesting placebo (PBO) or 20 mg of MEM. Subjective ratings are seen in millimeters, based on pencil marks on a 100 mm anchored visual analog scale (VAS). No changes reached statistical significance; consistent with this negligible subjective experience of MEM, only 60% of the AD subjects correctly identified the pill that they had taken on the test day that they received MEM, and on the day that they received placebo. We previously detected elevated “happy” ratings in response to this dose of MEM in individuals with schizophrenia (Swerdlow et al., 2015) and in young adult HCS (Swerdlow et al., 2009).

2.3. Correlations of experimental measures acquired on T1 and T2 are seen in Table S3. These findings suggest robust stability of most measures despite the fact that T1 and T2 involved different drug conditions (PBO vs. MEM (20 mg)) that might be expected to weaken test-retest correlations.

**Table S3**. Correlations of experimental measures on T1 and T2 in AD subjects. Note that testing followed PBO or MEM (20 mg) on T1 and T2, so r values do not accurately reflect test-retest reliability.

| **Experimental Measure** | **r** | **p** |
| --- | --- | --- |
| RBANS Total Score | 0.94 | <0.0001 |
| MMN amplitude | 0.76 | <0.0001 |
| P3a amplitude | 0.44 | <0.026 |
| MMN latency | 0.21 | ns |
| P3a latency | 0.65 | 0.0003 |
| Evoked Power | 0.79 | <0.0001 |
| Inter-trial Coherence (ITC) | 0.85 | <0.0001 |
| %PPI (60 ms) | 0.08 | ns |
| E/I Index | 0.82 | <0.0001 |

2.4. Table S4 shows rmANOVA results comparing changes in GDS (A) and NPI-Q (B) among patients with low vs. high sensitivity (“median split”) to acute MEM challenge (20 mg) in various experimental measures. With one exception (acute increases in P3a latency associated with reduced NPI-Q scores; p<0.02), low vs. high sensitivity to acute MEM challenge did not predict differential clinical responses (for GDS or NPI-Q) in AD subjects over 24 weeks of MEM treatment.

**Table S4.** Comparison of clinical gains (GDS (A), NPI-Q (B)) after 8, 16 and 24 weeks of MEM treatment in groups of AD subjects defined by low vs. high (median split) sensitivity to changes in experimental measures after acute “challenge” with MEM (20 mg).

**A. GDS**

| **Factor:** |  | **Median Split** |  |  | **Week** |  |  | **Split x Week** |  |
| --- | --- | --- | --- | --- | --- | --- | --- | --- | --- |
| **Measure** | **F** | **df** | **p** | **F** | **df** | **p** | **F** | **df** | **p** |
| RBANS total | 0.22 | 1,20 | ns | 0.34 | 2,40 | ns | 0.72 | 2,40 | ns |
| %PPI (60 ms) | 1.48 | 1,16 | ns | 0.24 | 2,32 | ns | 0.26 | 2,32 | ns |
| MMN amplitude | 0.10 | 1,19 | ns | 0.93 | 2,38 | ns | 0.57 | 2,38 | ns |
| P3a amplitude | 0.31 | 1,19 | ns | 0.45 | 2,38 | ns | 0.17 | 2,38 | ns |
| MMN latency | 0.30 | 1,19 | ns | 0.34 | 2,38 | ns | 0.83 | 2,38 | ns |
| P3a latency | 0.40 | 1,19 | ns | 0.34 | 2,38 | ns | 0.94 | 2,38 | ns |
| Evoked Power | 0.02 | 1,18 | ns | 0.46 | 2,36 | ns | 2.13 | 2,36 | ns |
| ITC | 0.01 | 1,18 | ns | 0.32 | 2,36 | ns | 1.62 | 2,36 | ns |
| E/I Index | 0.91 | 1,19 | ns | 0.40 | 2,38 | ns | 0.34 | 2,38 | ns |

**B. NPI-Q Severity Score**

| **Factor:** |  | **Median Split** |  |  | **Week** |  |  | **Split x Week** |  |
| --- | --- | --- | --- | --- | --- | --- | --- | --- | --- |
| **Measure** | **F** | **df** | **p** | **F** | **df** | **p** | **F** | **df** | **p** |
| RBANS total | 0.19 | 1,20 | ns | 1.15 | 2,40 | ns | 0.95 | 2,40 | ns |
| %PPI (60 ms) | 0.45 | 1,16 | ns | 1.23 | 2,32 | ns | 2.07 | 2,32 | ns |
| MMN amplitude | 0.46 | 1,19 | ns | 0.49 | 2,38 | ns | 0.83 | 2,38 | ns |
| P3a amplitude | 0.16 | 1,19 | ns | 0.56 | 2,38 | ns | 0.16 | 2,38 | ns |
| MMN latency | 0.14 | 1,20 | ns | 1.12 | 2,40 | ns | 0.26 | 2,40 | ns |
| P3a latency | 6.35 | 1,20 | **<0.02** | 1.15 | 2,40 | ns | 0.84 | 2,40 | ns |
| Evoked Power | 1.23 | 1,18 | ns | 0.60 | 2,36 | ns | 1.62 | 2,36 | ns |
| ITC | 1.67 | 1,18 | ns | 0.80 | 2,36 | ns | 2.35 | 2,36 | ns |
| E/I Index | 1.12 | 1,19 | ns | 0.60 | 2,38 | ns | 0.49 | 2,38 | ns |

2.5. Table S5 shows correlations coefficients (r) of: (A) acute MEM effects (20 mg) on experimental measures; or (B) baseline (BL) levels of measures, vs. changes in GDS score after treatment weeks 8, 16 and 24. Across all regressions, none identified correlations that reached statistical significance.

**Table S5.** Correlations (r) of acute MEM effect on experimental measures (A) or baseline (BL) levels of measures (B) vs. change in **GDS score** after weeks 8, 16 and 24 of MEM treatment (10 mg bid)

| **A. MEM effect on:** | GDS, Wk 8 - BL | GDS, Wk 16 - BL | GDS, Wk 24 - BL |
| --- | --- | --- | --- |
| RBANS total score | -0.17 | 0.27 | 0.26 |
| %PPI, 60 ms interval | -0.30 | -0.18 | -0.17 |
| MMN amplitude | 0.15 | -0.01 | -0.09 |
| P3a amplitude | -0.37 | -0.20 | -0.21 |
| MMN latency | -0.21 | 0.02 | -0.05 |
| P3a latency | 0.10 | 0.24 | 0.14 |
| Evoked power | -0.23 | -0.12 | -0.13 |
| Inter-trial coherence | 0.16 | 0.36 | 0.28 |
| E/I Index | 0.08 | 0.12 | -0.10 |
| **B. Baseline levels of:** |  |  |  |
| RBANS total score | -0.03 | -0.12 | 0.00 |
| %PPI, 60 ms interval | 0.02 | 0.18 | 0.12 |
| MMN amplitude | 0.01 | 0.16 | 0.09 |
| P3a amplitude | 0.05 | 0.32 | 0.21 |
| MMN latency | 0.05 | 0.02 | 0.12 |
| P3a latency | 0.10 | 0.05 | 0.15 |
| Evoked power | 0.05 | -0.05 | -0.11 |
| Inter-trial coherence | -0.02 | -0.02 | -0.11 |
| E/I Index | -0.09 | 0.30 | 0.33 |

2.6. Table S6 shows correlations coefficients (r) of: (A) acute MEM effects (20 mg) on experimental measures; or (B) baseline (BL) levels of measures, vs. changes in NPI-Q score after treatment weeks 8, 16 and 24. Across all regressions, only one identified a correlation that reached statistical signifi-cance (acute MEM-induced gains in E/I Index associated with *worsening* NPI-Q scores at week 16).

**Table S6**. Correlations (r) of acute MEM effect on experimental measures (A) or baseline (BL) levels of measures (B) vs. change in **NPI-Q score** after weeks 8, 16 and 24 of MEM treatment (10 mg bid)

| **A. MEM effect on:** | Wk 8 - BL | Wk 16 - BL | Wk 24 - BL |
| --- | --- | --- | --- |
| RBANS total score | 0.03 | -0.20 | 0.09 |
| %PPI, 60 ms interval | -0.01 | -0.28 | -0.33 |
| MMN amplitude | 0.15 | -0.13 | 0.17 |
| P3a amplitude | 0.33 | 0.02 | -0.06 |
| MMN latency | -0.25 | -0.06 | -0.02 |
| P3a latency | -0.05 | 0.13 | 0.15 |
| Evoked power | -0.33 | -0.30 | -0.21 |
| Inter-trial coherence | 0.13 | 0.19 | 0.21 |
| E/I Index | 0.17 | 0.45, p=0.03 | 0.37 |
| **B. Baseline levels of:** |  |  |  |
| RBANS total score | 0.08 | 0.06 | 0.12 |
| %PPI, 60 ms interval | -0.19 | 0.06 | 0.11 |
| MMN amplitude | -0.19 | 0.40 | 0.10 |
| P3a amplitude | -0.29 | -0.19 | 0.05 |
| MMN latency | 0.29 | 0.06 | 0.08 |
| P3a latency | 0.27 | 0.34 | 0.11 |
| Evoked power | 0.07 | -0.02 | -0.09 |
| Inter-trial coherence | 0.02 | 0.01 | -0.09 |
| E/I Index | 0.21 | -0.07 | -0.11 |

2.7. Exploratory analyses assessed the effects of several variables on sensitivity to clinical change across 24 weeks of open-label MEM treatment. As seen in Table S7, MEM’s impact on the primary outcome measure, ADAS-cog score, did not differ based on subject age, sex, illness duration, baseline MoCA, MMSE or ADAS-cog scores, concomitant use of an AChE inhibitor, or other subject characteristics at study entry. Similar results were seen with the secondary outcome measures, GDS and NPI-Q (data not shown).

**Table S7.** Exploratory analyses: Comparison of clinical gains (ADAS-cog) after 8, 16 and 24 weeks of MEM treatment in groups of AD subjects defined by several categorical variables.

| **Factor:** |  | **Group** |  |  | **Week** |  |  | **Group x Week** |  |
| --- | --- | --- | --- | --- | --- | --- | --- | --- | --- |
| **Variable** | **F** | **df** | **p** | **F** | **df** | **p** | **F** | **df** | **p** |
| Age (median split) | 0.40 | 1,20 | ns | 0.86 | 2,40 | ns | 0.13 | 2,40 | ns |
| sex | 1.67 | 1,20 | ns | 1.00 | 2,40 | ns | 3.42 | 2,40 | <0.045 |
| Illness duration* | 0.00 | 1,20 | ns | 0.88 | 2,40 | ns | 0.54 | 2,40 | ns |
| Age symptom onset* | 0.40 | 1,20 | ns | 0.86 | 2,40 | Ns | 0.13 | 2,40 | ns |
| Baseline MoCA* | 0.11 | 1,20 | ns | 0.93 | 2,40 | ns | 1.73 | 2,40 | ns |
| Baseline MMSE* | 0.08 | 1,20 | ns | 0.91 | 2,40 | ns | 1.19 | 2,40 | ns |
| Baseline ADAS-cog* | 0.00 | 1,20 | ns | 0.88 | 2,40 | ns | 0.59 | 2,40 | ns |
| Correct MEM pill guess | 0.75 | 1,20 | ns | 0.69 | 2,40 | ns | 0.51 | 2,40 | ns |
| AChE-Inhibitor (Y v. N) | 1.76 | 1,20 | ns | 0.58 | 2,40 | ns | 0.20 | 2,40 | ns |
| **APOEε4 allele (Y v. N)** | 0.13 | 1,20 | ns | 0.28 | 2,40 | ns | 1.12 | 2,40 | ns |
| rs10845840 ^#, 1^ | 0.03 | 1,19 | ns | 1.29 | 2,38 | ns | 0.41 | 2,38 | ns |
| rs727625 ^#, 2^ | 0.73 | 1,19 | ns | 0.86 | 2,38 | ns | 0.10 | 2,38 | ns |
| rs3739722 ^#, 3^ | 2.59 | 1,19 | ns | 0.67 | 2,38 | ns | 1.55 | 2,38 | ns |
| Mean MEM effect on: |  |  |  |  |  |  |  |  |  |
| heart rate* | 0.04 | 1,20 | ns | 0.89 | 2,40 | ns | 0.69 | 2,40 | ns |
| “Happy”* | 1.50 | 1,20 | ns | 0.90 | 2,40 | ns | 0.96 | 2,40 | ns |
| “Drowsy”* | 2.11 | 1,20 | ns | 0.92 | 2,40 | ns | 1.45 | 2,40 | ns |
| “Anxious”* | 2.76 | 1,20 | ns | 0.88 | 2,40 | ns | 0.62 | 2,40 | ns |

* Median Split; # (CC vs. (CT, TT); 1. GRIN 2A; 2. GRIN 2B; 3. GRIN 3A (see below, Figure S3(B-D))

**Figure S3.** Change from baseline ADAS-cog scores over 24 weeks of MEM treatment did not differ among AD subjects based on the presence of at least one APOE **ε4 allele (A) or at least one thymine allele (T) for polymorphisms at the GRIN2A, GRIN2B or GRIN3A sites (B-D, respectively).**

Pharmacogenetic studies in AD have tended to focus on AChE inhibitors (cf., Cacabelos 2020) and have reported that APOE ε4 carriers show a diminished response to these drugs. Here, carrying one or two APO ε4 alleles was not associated with differential clinical response to MEM (change in ADAS-cog) (Table S7; Figure S3A). Clinical response to MEM also did not differ among subjects distinguished by glutamate receptor subtype polymorphisms (GRIN 2A, GRIN 2B, GRIN 3A); specifically, no differences in ADAS-cog response were detected between individuals carrying at least one T allele versus no T allele (CC genotype) for any of these polymorphisms (Table S7; Figure S3B-D). Mutations in these genes are associated with differential MEM sensitivity in other neurological disorders (Lesca et al., 2013; Li et al., 2016; Pierson et al., 2014).

**3. Supplemental Discussion**

3.1. Experimental measures in AD subjects vs. HCS

While it was not the primary focus of this study, it is worth commenting on the differences (or lack thereof) identified in the experimental measures in AD vs. HCS groups. As noted below, the literature on these measures in AD is mixed, with deficits identified in some but not all reports:

3.1.2. PPI: In this study, deficits in PPI were identified in AD subjects at 60 ms prepulse intervals. Conceivably, PPI deficits in AD might be mediated via pathology in entorhinal cortex, a region impacted in AD and implicated in the regulation of PPI in both humans (Kumari et al., 2004, 2008) and rodents (Swerdlow et al., 2001; Goto et al. 2002, 2004). However, the literature on PPI in AD subjects is mixed, with deficits reported by some groups (e.g., Ueki et al., 2006; Aziz et al. 2019) but not by others (e.g., Hejl et al., 2004; Salem et al. 2011; cf. Jafari et al., 2020). Deficits in the present study were detected only at the 60 ms prepulse interval, and not at either shorter (10-30 ms) or longer (120 ms) intervals. The 60 ms interval is at the threshold of conscious detection and hence pre-attentive vs. attentionally-sensitive inhibition; in our experience, this 60 ms interval has been most sensitive to deficits in other patient populations (e.g., Swerdlow et al., 2018) and most sensitive to MEM-enhanced PPI (Swerdlow et al. 2016, 2023). Studies of animal models relevant to AD have identified both PPI deficits, and “rescue” of PPI by proposed therapeutic agents (e.g., Price et al., 2012; Koppel et al., 2014; Krivinko et al., 2022).

Prolonged peak startle latency in AD subjects has also been reported by at least two groups (Salem et al., 2011; Aziz et al., 2019), though in one report these deficits were not evident after age correction (Hejl et a., 2004). We did not detect prolonged peak startle latency in AD vs. HCS subjects in the present study (F=0.92, df 1,48, ns).

3.1.3. MMN / P3a: As with PPI, the literatures on MMN and P3a in AD are mixed. While differences across studies might reflect the use of different stimulus characteristics, the simplest interpretation is that MMN amplitude is reduced in aging but that there are no clear changes associated with AD per se (cf. Vecchio and Määttä, 2011). The present study did not detect abnormal MMN amplitude or latency in AD subjects vs. HCS of comparable age. Our failure to detect group differences in MMN amplitude might reflect a Type II error: given the small-to-medium effect size (d = 0.48; p<0.10), the sample size (n’s = 27 and 24 for AD and HCS groups, respectively) provides power of about 0.40 to detect significant group differences at alpha = 0.05.

Prolonged P3a latency in AD subjects was detected in the present study, consistent with some (Frodl et al., 2002; Juckel et al., 2008) but not all reports (Yamaguchi et al., 2000).

3.1.4. ASSR (power and coherence): A limited number of studies report ASSR findings in AD subjects, though at least two report *increased* power in AD vs. HCS (Osipova et al., 2006; Van Deursen et al., 2011). In contrast to these reports, we detected reductions in both ASSR power and inter-trial coherence in AD subjects vs. HCS. The high levels of test-retest reliability with these measures from the present study (r = 0.79-0.85) provides some confirmation of the robustness of the present findings. Future studies will be needed to determine the basis for these discrepant findings.

3.1.5. E/I Index: One hypothesis for the pathophysiology of AD suggests that, as a result of the accumulation and interaction of amyloid‐beta and tau proteins, AD brain tissue becomes hyperexcitable and hypersynchronized, causing neurotoxic injury. This hyperexcitability is inferred from shifts in the aperiodic component of the neural power spectrum, often referred to as the ‘1/f slope’, though there is disagreement regarding the role of changes in this aperiodic slope as a causal feature in AD pathology (van Nifterick et al., 2023; Kopčanová et al., 2024). A range of neuroimaging, electrophysiological, gene expression and neurochemical techniques have been used to detect excessive excitation in brain tissue in AD and related preclinical models (cf. van Nifterick et al., 2023). The present study extracted the 1/f slope from the EEG signal and detected no difference in E/I balance between AD and HCS groups. There are several possible explanations for this apparent discrepancy between the present findings and the hypothesized E/I balance abnormalities in AD, including differences in study designs, resting state vs. oddball background, drug challenges (or lack thereof) and signal preprocessing.

3.2. No clear “biomarker” of MEM therapeutic sensitivity

The primary hypothesis of this study was that the magnitude of change in one or more of 9 experimental measures after acute MEM challenge would predict (serve as a “biomarker” for) sensitivity to the therapeutic impact of MEM over a 24-week trial. Two approaches were used to assess such predictive ability: 1) a categorical approach, in which clinical changes were compared across AD subjects divided into two groups based on low vs. high sensitivity to MEM on each experimental measure; and 2) a continuous approach, that assessed the correlation between the arithmetic change in each experimental measure after acute MEM challenge and the arithmetic change in symptom level after 8, 16 or 24 weeks of MEM treatment.

The categorical approach, supported by rmANOVA, identified no experimental measure for which low vs. high sensitivity to acute MEM challenge was associated with significant differences in clinical outcome. The continuous approach, via simple regression analyses, identified two examples in which the amount of change of a measure after acute MEM challenge was significantly correlated with the change in symptoms after 8, 16 or 24 weeks of MEM treatment: 1) acute change in MMN latency correlated significantly with changes in ADAS-cog measures after 8 weeks (r=0.53, p<0.008) and 24 weeks of treatment (r=0.45, p<0.04), with a similar trend at 16 weeks (r=0.37, ns); this correlation remained robust when ADAS-cog gains were averaged across the 3 test days (r= 0.52, p<0.017). 2) MEM effects on ASSR coherence correlated significantly with changes in ADAS-cog measures after 24 weeks of treatment (r= - 0.48, p<0.03). In both cases, the “direction” of the correlation was consis-tent with the proposed predictive value: reduced (“healthier”) MMN latency after acute MEM challenge was associated with cognitive gains during MEM treatment and increased (“healthier”) ASSR coherence after acute MEM was associated with cognitive gains during MEM treatment (Figures 4A and 4B, respectively). Nonetheless, significant correlations detected in 2 (MMN latency; ASSR coherence) out of 9 measures, or even in 3 (MMN latency at weeks 8 and 24, and ASSR coherence at week 24) out of 27 correlations (9 measures x 3 time points), might reflect chance events.

There are several possible explanations for the inability to detect robust relationships between acute MEM sensitivity in experimental measures and sensitivity to MEM’s therapeutic impact over 24 weeks:

3.2.1. Wrong experimental measures: This choice of measures was based on findings from studies in substantially younger, non-AD subjects, in which acute MEM challenge resulted in a “neurophysiological signal” – e.g., increased PPI (Swerdlow et al., 2016) or ASSR power and coherence (Light et al., 2017) – that provided evidence of MEM’s acute neuroactivity. A signal that MEM is acutely neuroactive indicates that MEM is reaching and impacting brain tissue, but such impact might or might not have relevance to its therapeutic activity.

3.2.2. Wrong time course: Since MEM’s therapeutic effects were based on outcome measures at 8, 16 and 24 weeks, its “neuroactivity” based on changes in experimental measures within hours after a single dose might not be useful for “predicting” therapeutic sensitivity weeks later. The timing of experimental measures after acute MEM challenge was based on both studies of MEM absorption (Sonkusare et al. 2005) and of MEM effects on startle and EEG measures studied here (Swerdlow et al. 2016; Light et al., 2017; Molina et al., 2020), but there is no certainty that these time points post-MEM administration would be most useful for predicting MEM’s therapeutic impact. Of course, a “predictive measure” is only useful if it provides a signal of bioactivity well in advance of the therapeutic impact. It is also conceivable that the study timeline was too short to detect MEM’s full therapeutic effects. However, the 24-week time course for clinical response was selected based on published reports of MEM’s superior effectiveness vs. placebo detected by week 8, and persisting through week 24 (Peskind et al, 2006; Pomara et al., 2007).

3.2.3. Underpowered: The study may have lacked adequate power to detect a relationship between MEM’s acute effects on experimental measures and its therapeutic properties. This is a fair critique, since median splits yielded small groups (n’s = 11-12 AD subjects per cell for “low” vs. “high” MEM sensitivity). Inspection of the statistics for main effects of sensitivity (median split) for the primary and secondary outcomes reveals a preponderance of F values that were less than 1.0 (18/27), suggesting that data from larger samples might not have substantively changed most of these results. Furthermore, even the “larger” F-values reflected relationships that were as often “negative” (greater acute MEM sensitivity associated with less MEM therapeutic sensitivity) as they were “positive”. This last observation leads to the next possible explanation for the failure to detect the predicted relationship between acute experimental vs. therapeutic MEM sensitivity: *perhaps acute MEM sensitivity was defined incorrectly in these studies.*

3.2.4. Wrong metric of “sensitivity”: Acute MEM sensitivity was defined by a difference score (MEM minus placebo) for each experimental measure, and a median split of this difference score was used to define subgroups with “low” vs. “high” MEM sensitivity (see Figure S4, “Model 1”). However, one could argue that difference scores close to zero might best reflect low MEM sensitivity, while extreme positive *and* negative difference scores (or large *absolute values* of difference scores) might best reflect high MEM sensitivity (see Figure S4, “Model 2”). To test this hypothesis, rmANOVAs were repeated comparing primary outcome measures in AD subgroups defined by: 1) being in the *lowest or highest quartile* of acute MEM difference scores vs. 2) median + one quartile of the acute MEM difference score. Findings from this analysis are seen in Table S8 for ADAS-cog analyses and with one exception (RBANS total score) revealed no significant predictors of MEM’s therapeutic impact.

**Figure S3.** Two different models (metrics) for defining MEM sensitivity of experimental measures, using the example of ASSR inter-trial coherence (ITC). For both models, acute MEM sensitivity was based on a difference score (MEM minus PBO) for ITC values. In Model 1, “high” vs. “low” sensitivity was defined based on a median split of this difference score. In Model 2, difference scores close to zero (0 + one quartile) define “low” MEM sensitivity, while extreme quartiles of positive and negative difference scores (or large absolute values of difference scores) define “high” MEM sensitivity.

**Table S8.** RmANOVA results of ADAS-cog scores in groups with “low” vs. “hi” MEM sensitivity as per Figure S8, “Model 2”:

| **Factor:** |  | **Sensitivity group** |  |  | **Week** |  |  | **Group x Week** |  |
| --- | --- | --- | --- | --- | --- | --- | --- | --- | --- |
| **Measure** | **F** | **df** | **p** | **F** | **df** | **p** | **F** | **df** | **p** |
| RBANS total | 6.11 | 1,20 | **<0.023** | 0.89 | 2,40 | ns | 0.77 | 2,40 | ns |
| %PPI (60 ms) | 0.54 | 1,16 | ns | 1.20 | 2,32 | ns | 0.32 | 2,32 | ns |
| MMN amplitude | 0.23 | 1,19 | ns | 0.47 | 2,38 | ns | 0.54 | 2,38 | ns |
| P3a amplitude | 1.97 | 1,19 | ns | 0.41 | 2,38 | ns | 0.14 | 2,38 | ns |
| MMN latency | 1.30 | 1,19 | ns | 0.41 | 2,38 | ns | 0.24 | 2,38 | ns |
| P3a latency | 2.76 | 1,19 | ns | 0.40 | 2,38 | ns | 0.41 | 2,38 | ns |
| Evoked Power | 0.28 | 1,18 | ns | 0.37 | 2,36 | ns | 0.61 | 2,36 | ns |
| Inter-trial coherence | 0.11 | 1,18 | ns | 0.37 | 2,36 | ns | 3.74 | 2,36 | <0.035 |
| E/I Index | 0.00 | 1,20 | ns | 0.86 | 2,40 | ns | 0.01 | 2,40 | ns |

3.2.5. Too healthy: It is conceivable that the failure to identify a “biomarker” predicting clinical sensitivity to MEM reflected the fact that our AD subjects were relatively mild in their impairment (baseline ADAS-cog range 8.33 – 39.33). ADAS-cog total scores for AD group subjects averaged roughly 20 across the 24-week study, less impaired than past studies demonstrating MEM efficacy in mild-to-moderate severity AD (e.g. mean baseline ADAS-cog = 27.2; Peskind et al., 2006). There is some evidence that MEM is most effective in AD patients with more severe symptoms such as agitation and psychosis (e.g., Wilcock et al., 2008); no subjects in the present cohort exhibited these symptoms. To assess the impact of baseline symptom severity on the “predictability” of MEM sensitivity in this study, we repeated the rmANOVAs with low vs. high baseline ADAS-cog score as a grouping factor (data not shown). These analyses confirmed that the acute impact of MEM on the experimental measures was an equally insensitive predictor of MEM therapeutic sensitivity among individuals with mild or moderate ADAS-cog impairment (mean ADAS-cog scores = 12.28 and 27.83, respectively; main effect of severity: F=0.00, df 1,21, ns).

3.2.6. Compressed range: The failure of acute MEM effects on experimental measures to predict MEM therapeutic sensitivity might result from an insufficient heterogeneity in MEM effects on experimental measures. In other words, a compressed range of centralized values might have resulted in median split groupings that did not reflect meaningful differences in acute MEM sensitivity. Inspection of the distributional properties of MEM effects on these experimental measures (Figure S4) reveals some instances in which this distribution is substantial and for which median splits should provide a meaningful comparison of groups with low vs. high MEM sensitivity (e.g., RBANS, MMN and P3a amplitude, P3a latency, ASSR coherence and E/I Index) and other instances where a compressed central distribution, with perhaps a limited number of extreme values may have resulted in less meaningful low vs. high sensitivity group comparisons (e.g., PPI, MMN latency and ASSR evoked power). Importantly, measures yielding these different types of distributions were equally ineffective at predicting MEM’s therapeutic impact.

**Figure S4.** Distributions of MEM effects (value after MEM minus value after PBO) for each of the 9 primary experimental measures: A. RBANS total score; B. %PPI (60 ms); C. MMN amplitude; D. P3a amplitude; E. MMN latency; F. P3a latency; G. ASSR power; H. ASSR inter-trial coherence (ITC); I. E/I index. Conceivably, compressed distributional properties for any given measure might obscure detection of significant group differences based on categorical (median splits) or continuous (regression) analyses. Scatter plots show relatively broadly distributed for most measures, with others exhibiting scores compressed near zero. Importantly, these distributional properties did not seem to be associated with a measure’s ability to predict a therapeutic response to MEM.

3.2.7. Interactions: This study tested the ability of acute MEM effects on 9 experimental measures, along with some exploratory clinical, demographic and genetic variables, to predict clinical sensitivity to MEM in AD subjects. In each case, the predictive value was assessed for individual measures or variables; it is conceivable that predictive value would be detected only in the combination or interaction of these measures or variables. Testing such a hypothesis would require a substantially larger study sample; more importantly, an outcome in which combinations of measures / variables are required to predict MEM sensitivity for a “personalized” intervention would have limited clinical utility.

3.2.8. Relatively weak effects of MEM: MEM was used to test two drug effects in this study – acute effects on 9 experimental measures, and sustained effects on 3 clinical measures – and in both cases, these effects of MEM appeared relatively weak. These weak effects might have obscured our ability to detect the predictive relationships being investigated in this study. Acute MEM “bioactivity” can be inferred from its effects on several measures in this study, including some that were expected (e.g., MEM-induced increases in PPI, ASSR power and inter-trial coherence) and two that were unexpected (MEM-induced decrease in P3a amplitude and latency). All of these effects were relatively week, with small-to-medium effect sizes of 0.46, 0.56, 0.36, 0.23 and 0.21, respectively, and only 2 reached two-tailed statistical significance. While these measures collectively support the notion that the 20 mg dose of MEM was neuroactive (consistent with past reports from our group and others, e.g., Korostenskaja et al., 2007; Noorman et al., 2025), subjective measures also did not detect any acute effects of MEM challenge. Regarding the effects of MEM after sustained administration, based on the open-label design, it is difficult to infer the magnitude of the therapeutic MEM effect in AD. On the one hand, some therapeutic activity is suggested by the fact that there was no significant clinical deterioration in AD subjects as a group over their 24 weeks of treatment; on the other hand, there was also no improvement in ADAS-cog scores as a group, such as that reported in AD subjects in some previous studies (Peskind et al., 2006; Pomara et al., 2007). Thus, the paucity of predictive relationships between the acute and sustained effects of MEM in this study might reflect the fact that both of these MEM effects were relatively weak. Conceivably, this issue might be resolved with a larger sample size, or alternatively, with more potent pharmacologic analogs of memantine.

**References**

Aziz, V., 2019. Deficit in sensorimotor gating in Alzheimer’s disease (AD): measuring prepulse inhibition (PPI) as a measure of liability to AD Ann. Psychiatry Clin. Neurosci. 2, pp. 1-5.

Bigdely-Shamlo, N., Mullen, T., Kothe, C., et al., 2015. The PREP pipeline: standardized preprocessing for large-scale EEG analysis Front. Neuroinform. 9, 16.

Cacabelos, R., 2020. Pharmacogenetic considerations when prescribing cholinesterase inhibitors for the treatment of Alzheimer's disease. Expert Opin Drug Metab Toxicol. 16(8):673-701.

Donoghue, T., Haller, M., Peterson, E.J., et al. 2020. Parameterizing neural power spectra into periodic and aperiodic components. Nat Neurosci. 23(12):1655-1665.

Frodl, T., Hampel, H., Juckel, G., et al., 2002. Value of event-related P300 subcomponents in the clinical diagnosis of mild cognitive impairment and Alzheimer’s disease. Psychophysiology. 39(2), 175–181.

Goto, K., Ueki, A., Iso, H., et al., 2002. Reduced prepulse inhibition in rats with entorhinal cortex lesions. Behav. Brain Res. 134, 201–207.

Goto, K., Ueki, A., Iso, H., et al., 2004. Involvement of nucleus accumbens dopaminergic transmission in acoustic startle: observations concerning prepulse inhibition in rats with entorhinal cortex lesions. Psychiatry Clin. Neurosci. 58, 441–445.

Hejl, A.M., Glenthøj, B., Mackeprang, T., et al., 2004. Prepulse inhibition in patients with Alzheimer’s disease. Neurobiol. Aging. 25, 1045-1050.

Jafari, Z., Kolb, B.E., Mohajerani, M.H., 2020, Prepulse inhibition of the acoustic startle reflex and P50 gating in aging and Alzheimer's disease. Ageing Res. Rev. 59, 101028.

Juckel, G., Clotz, F., Frodl, T., et al., 2008. Diagnostic usefulness of cognitive auditory event-related p300 subcomponents in patients with Alzheimers disease? J. Clin. Neurophysiol. 25(3), 147-152.

Kopčanová, M., Tait, L., Donoghue, T., et al. 2024. Resting-state EEG signatures of Alzheimer's disease are driven by periodic but not aperiodic changes. Neurobiol Dis. 190:106380.

Koppel, J., Jimenez, H., Azose, M., et al., 2014, Pathogenic tau species drive a psychosis‐like phenotype in a mouse model of Alzheimer's disease. Behav. Brain Res. 275, 27‐33.

Korostenskaja, M., Nikulin, V.V., Kicic, D., et al., 2007. Effects of NMDA receptor antagonist memantine on mismatch negativity. Brain Res. Bull. 72(4-6), 275-283.

Krivinko, J.M., Erickson, S.L., MacDonald, M.L., 2022. Fingolimod mitigates synaptic deficits and psychosis-like behavior in APP/PSEN1 mice. Alzheimers Dement. (N Y). 8(1), e12324.

Kumari, V., Antonova, E., Zachariah, E., et al., 2005. Structural brain correlates of prepulse inhibition of the acoustic startle response in healthy humans. Neuroimage. 26, 1052–1058.

Kumari, V., Fannon, D., Geyer, M.A., et al., 2008. Cortical grey matter volume and sensorimotor gating in schizophrenia. Cortex. 44, 1206–1214.

Lesca, G., Rudolf, G., Bruneau, N., et al., 2013. GRIN2A mutations in acquired epileptic aphasia and related childhood focal epilepsies and encephalopathies with speech and language dysfunction. Nature Genet. 45, 1061-1066.

Li, D., Yuan, H., Ortiz-Gonzalez, X.R., et al., 2016. GRIN2D recurrent de novo dominant mutation causes a severe epileptic encephalopathy treatable with NMDA receptor channel blockers. Am. J. Hum. Genet. 99, 802-816.

Light, G.A., Zhang, W., Joshi, Y.B., et al., 2017. Single-Dose memantine improves cortical oscillatory response dynamics in patients with schizophrenia. Neuropsychopharmacology. 42, 2633-2639.

Ma, J., Mufti, A., Stan Leung, L., 2015, Effects of memantine on hippocampal long-term potentiation, gamma activity, and sensorimotor gating in freely moving rats. Neurobiol. Aging. 36(9), 2544-2554.

Molina, J.L., Voytek, B., Thomas, M.L., et al., 2020. Memantine Effects on Electroencephalographic Measures of Putative Excitatory/Inhibitory Balance in Schizophrenia. Biol. Psychiatry Cogn. Neurosci. Neuroimaging. 5, 562-568.

Noorman, S., Stein, T., Zantvoord, J., et al., 2025. A causal role of the NMDA receptor in recurrent processing during perceptual integration. eLife. Jun 18;13:RP100530.

Osipova, D., Pekkonen, E., Ahveninen, J., 2006. Enhanced magnetic auditory steady-state response in early Alzheimer's disease. Clin. Neurophysiol. 117, 1990-1995.

Peskind, E.R., Potkin, S.G., Pomara, N., et al., 2006. Memantine treatment in mild to moderate Alzheimer disease: a 24-week randomized, controlled trial. Am. J. Geriatr. Psychiatry. 14, 704-715.

Perriol, M.P., Dujardin, K., Derambure, P., et al., 2005. Destee Disturbance of sensory filtering in dementia with Lewy bodies: comparison with Parkinson’s disease dementia and Alzheimer’s disease J. Neurol. Neurosurg. Psychiatry. 76, pp. 106-108.

Pierson, T.M., Yuan, H., Marsh, E.D., et al., 2014. GRIN2A mutation and early-onset epileptic encephalopathy: personalized therapy with memantine. Ann. Clin. Transl. Neurol. 1, 190-198.

Pion-Tonachini, L., Kreutz-Delgado, K., Makeig, S. 2019. ICLabel: An automated electroencephalographic independent component classifier, dataset, and website. Neuroimage. 198:181-197.

Pomara. N., Ott, B.R., Peskind, E., et al., 2007. Memantine treatment of cognitive symptoms in mild to moderate Alzheimer disease: Secondary analyses from a placebo-controlled randomized trial. Alzheimer Dis. Assoc. Disord. 21, 60-64.

Price, D.L., Bonhaus, D.W., Mcfarland, K., 2012. Pimavanserin, a 5‐HT2A receptor inverse agonist, reverses psychosis‐like behaviors in a rodent model of Alzheimer's disease. Behav. Pharmacol. 23(4), 426-433.

Salem, L.C., Hejl, A.M., Garde, E., 2011. White matter hyperintensities and prepulse inhibition in a mixed elderly population. Psychiatry Res. 194, pp. 314-318.

Sonkusare, S.K., Kaul, C.L., P Ramarao, P., 2005. Dementia of Alzheimer's disease and other neurodegenerative disorders--memantine, a new hope. Pharmacol. Res. 51(1), 1-17.

Swerdlow, N.R., Joshi, Y.B., Sprock, J., et al., 2023. Preliminary Evidence that Memantine Enhances Prepulse Effects on Startle Magnitude and Latency in Patients with Alzheimer's Disease. J. Alzheimers Dis. 91(1), 355-362.

Swerdlow, N.R., Bhakta, S., Chou, H.H., et al., 2016. Memantine Effects on Sensorimotor Gating and Mismatch Negativity in Patients with Chronic Psychosis. Neuropsychopharmacology. 41, 419-430.

Swerdlow, N.R., Light, G.A., Thomas, M.L., et al., 2018. Deficient prepulse inhibition in schizophrenia in a multi-site cohort: Internal replication and extension. Schizophr. Res. 198, 6-15.

Swerdlow, N.R., Hanlon, F.M., Henning, L., et al., 2001. Regulation of sensorimotor gating in rats by hippocampal NMDA: anatomical localization. Brain Res. 898(2), 195-203.

Ueki, A., Goto, K., Sato, N., et al., 2006. Prepulse inhibition of acoustic startle response in mild cognitive impairment and mild dementia of Alzheimer type. Psychiatry Clin. Neurosci. 60, 55-62.

van Deursen, J.A., Vuurman, E.F.P.M., van Kranen-Mastenbroek, V.H.J.M., et al., 2011. 40-Hz steady state response in Alzheimer's disease and mild cognitive impairment. Neurobiol. Aging. 32, 24-30.

van Nifterick, A.M., Mulder, D., Duineveld, D.J. et al. 2023. Resting-state oscillations reveal disturbed excitation–inhibition ratio in Alzheimer’s disease patients. Sci. Rep. 13, 7419.

Vecchio, F., Määttä, S., 2011. The use of auditory event-related potentials in Alzheimer's disease diagnosis. Int. J. Alzheimers Dis. 2011, 653173.

Wilcock, G.K., Ballard, C.G., Cooper, J.A., Loft, H., 2008. Memantine for agitation/aggression and psychosis in moderately severe to severe Alzheimer's disease: a pooled analysis of 3 studies. J. Clin. Psychiatry. 69(3), 341–348.

Yamaguchi, S., Tsuchiya, H., Yamagata, S., et al., 2000. Event-related brain potentials in response to novel sounds in dementia. Clinical Neurophysiology. 111(2), 195–203.
